# Effects of vacuum sealing drainage on the treatment of cranial bone-exposed wounds in rabbits

**DOI:** 10.1590/1414-431X20175837

**Published:** 2017-10-02

**Authors:** X.J. Chen, S. Liu, G.Z. Gao, D.X. Yan, W.S. Jiang

**Affiliations:** Department of Burn and Plastic Surgery, the 253rd Hospital of People's Liberation Army, Xincheng District, Hohhot, Inner Mongolia, China

**Keywords:** Cranial bone-exposed wound, Vacuum sealing drainage, Periosteum, Wound healing, Efficacy

## Abstract

This study was designed to assess the efficacy of vacuum sealing drainage (VSD) on skull exposure wounds in rabbits and to investigate the underlying mechanism of the process. Full-thickness excisional circular wounds 2×2 cm with or without periosteum involvement were created in 88 New Zealand white rabbits (mean body weight: 3.0±0.65 kg). Animals were randomly divided into 4 groups: periosteum-intact wounds treated with traditional dressing (p+control), periosteum-intact wounds treated with VSD (p+VSD), periosteum-lacking wounds treated with traditional dressing (p–control) and periosteum-lacking wounds treated with VSD (p–VSD). The wounds treated with traditional dressing were covered with Vaseline gauze, while VSD treatment was accompanied with continuous –120 mmHg pressure. Finally, wound tissues were harvested for analysis of hydroxyproline content and histologic detection. VSD hastened the wound healing process significantly (P<0.05) compared to the corresponding control groups. VSD alleviated the inflammation reaction, accelerated re-epithelialization and facilitated the organization of collagen fibers into neat rows. During the wound healing process, the hydroxyproline content increased overtime [i.e., postoperative days (POD) 7, POD 10 and POD 15] in all four groups, and it peaked in the p+VSD group. VSD also promoted angiogenesis via increasing number and quality of collagen. We concluded that VSD can promote healing in bone-exposed wounds via increasing hydroxyproline content and vessel density, reducing inflammatory responses and generating ordered collagen arrangement.

## Introduction

The number of patients with large-area soft tissue defects accompanied by bone exposure has been increasing due to frequently-occurring road accidents, high-energy explosives, deep burns, work-related injuries and tumor resection (1). Such wounds are often associated with severe avulsion, infection, and poor blood supply, therefore requiring a long healing time. The traditional wound dressing methods often cause complications such as wound infection, non-healing bone fracture, non-union, osteonecrosis, osteomyelitis and fistula formation. Serious infections can lead to amputation. Hence, it is critical to resolve certain problems, including prevention of pathologic microorganisms, temporary cover of wound surface, among others.

The vacuum sealing drainage (VSD) technique (2) has been widely applied for effectively treating diverse wound surfaces, especially for injuries that could not achieve debridement and coverage in a one-stage procedure. The technique involves usage of polyvinyl alcohol/alginate blend foam, drainage tube, semi-permeable membranes (3) and a negative pressure source. Gases generated from decomposition of necrotic tissues within the wound surface can smoothly infiltrate into outer space of the semi-permeable membrane, yet air and bacteria outside the membrane cannot enter the wound surface successfully. Nonetheless, the healthy skin around the wound surface can breathe in a normal way.

Overall, VSD can improve partial blood circulation, thus reducing edema and bacteria (4), promoting growth of granulation tissue and enhancing the healing rate. The inhibitory effects on bacteria reproduction could largely result from the negative pressure of VSD, which creates a relatively anoxic environment on the wound surface, without affecting the surrounding healthy tissues. Owing to these merits of VSD, surgeons have broadly accepted VSD for treatment of soft tissue trauma (1,5,6) and bone-exposed wounds (7–9). Nonetheless, few comprehensive analyses have investigated the effectiveness and inherent mechanism of VSD treatment for bone-exposed wounds. Thus, we used the rabbit skull-exposed wound model to compare the efficacy of VSD with traditional dressing therapy by histological and vascular density analyses, and hydroxyproline content.

## Material and Methods

### Grouping of animals

A total of 88 New Zealand white rabbits (mean body weight: 3.0±0.65 kg) were provided by the 253rd Hospital of People's Liberation Army of China (PLA). The rabbits were randomly divided into 4 groups of periosteum-intact traditional dressing therapy (p+control), periosteum-intact VSD-treated (p+VSD), periosteum-lacking traditional dressing therapy (p–control), and periosteum-lacking VSD-treated (p–VSD). The experiment protocol for this study was approved by the Ethics Committee for Animal Research of PLA 253rd Hospital SCXK-(Huhhot) 2015-0021.

### Model preparation

After anesthetizing the rabbits through intra-peritoneal injection of pentobarbital sodium (concentration: 30 mg/kg), their heads were shaved and smeared with sodium sulfide (6%). Subsequently, rabbits in the prone position were disinfected with 75% ethyl alcohol. Specific operations relevant to successive removal of skin, subcutaneous tissue and galea aponeurotica, as well as exposure of periosteum were performed according to a previously reported investigation (10). Finally, full-thickness wounds (2×2 cm) were created in either periosteum-intact or periosteum-lacking forms.

In the traditional dressing therapy group, wounds were covered with Vaseline gauze every other day. In the VSD group, VSD foam (Weisidi Medical Technology Corporation, China) was cut >3 mm beyond the wound margins. Then, we inserted a porous silica gel tube around the foam, and connected it to a negative pressure device (Weisidi Medical Technology Corporation). The negative pressure source was switched on, and a biological semi-permeable membrane (Smith & Nephew Corporation, UK) was pasted. The device was set to negative pressure (i.e., –120 mmHg) continuously for 16 days (i.e., the 15th postoperative day (POD) since the surgery), and the seal of the device was strictly checked every 6 h. Vacuum sealing drainage was conducted daily, and healing was measured over time. The wound tissues were harvested on POD 7, 10, and 15 from at least three animals for the detection of hydroxyproline content and histological analysis.

### Gross assessment of wounds and histological analysis

Daily photographs were taken to visually track wound healing progress. The wounds were traced on transparent graph paper and then their sizes were analyzed with Image-Pro Plus 6.0 software (Version X; Media Cybernetics, USA). The rate of closure was calculated as daily change of the wound area relative to the initial wound area. Wound healing time was defined as the time required for epithelial healing.

For histologic analyses, wounds were harvested within a 2–3-mm rim from rabbits that were euthanized at POD 7, 10, and 15. Half of the wounds were placed in 10% formalin, while the other half were frozen in liquid nitrogen and stored at –80°C. The formalin-fixed wounds were embedded in paraffin and cut into 5-μm sections, then placed onto glass slides and stained with hematoxylin and eosin (H&E) (Sigma-Aldrich Corporation, Germany). Each slide was measured objectively and automatically at least three times.

### Collagen quantification

The remaining tissues were stained with Masson's trichrome (Sigma-Aldrich) to visualize collagen fibers. As hydroxyproline is a basic constituent of collagen structure, its content can serve as indicator of collagen synthesis. Hydroxyproline contents were analyzed on POD 7, 10, and 15, in line with manufacturers' instructions of hydroxyproline analysis kit (Jiancheng Technology Corporation, China, catalog number: A030-2). To be specific, tissue samples were successively mixed with 10 mM CuSO_4_ (1 mL), 2.5 M NaOH (1 mL), and 6% H_2_O_2_ (1 mL). Subsequently, the mixture was incubated at 80°C for 5 min with frequent vigorous shaking, and then 3 M H_2_SO_4_ (4 mL) was added with agitation. Finally, after addition of 5% p–dimethyl amino benzaldehyde (2 mL), the mixed solution was incubated at 60°C for 15 min. The absorbance (optical density) was evaluated at 550 nm under the microplate reader (Tecan Genios Corporation, Australia).

### Measurement of microvascular density (MVD)

Since CD31 acts as a vascular endothelial marker, staining of CD31 (Santa Cruz Corporation, USA) could be reflective of MVD of the wounds. Two blinded investigators counted the stained blood vessels in intense vascular areas (i.e., hotspot) of the tissue section, according to the method described by Weidner et al. (11). Of note, any single endothelial cell or endothelial cell cluster that was stained yellow brown served as a capillary. The endothelial cells with clear boundaries were counted as a blood vessel, but the blood vessels with lumen size greater than the size of 8 red blood cells or muscular layer were not counted. Besides, vessels of 10 non-overlapping areas of the above-mentioned hotspot were visualized and counted under high-power magnification (200×), and the derived data was deemed as MVD of that tissue (10–12). The index (%) of MVD was calculated according to the following formula: MVD index (%) = count of positive cells / count of total cells × 100.

### Statistical analysis

The statistical analyses were performed using SPSS17.0 software (SPSS, USA). The measurement data are reported as means±SD, and statistical comparisons were performed using either unpaired Student's *t*-test or multivariate analysis of variance (ANOVA). The P value was considered statistically significant when it was less than 0.05.

## Results

### VSD therapy accelerated wound healing

**Figure 1.**
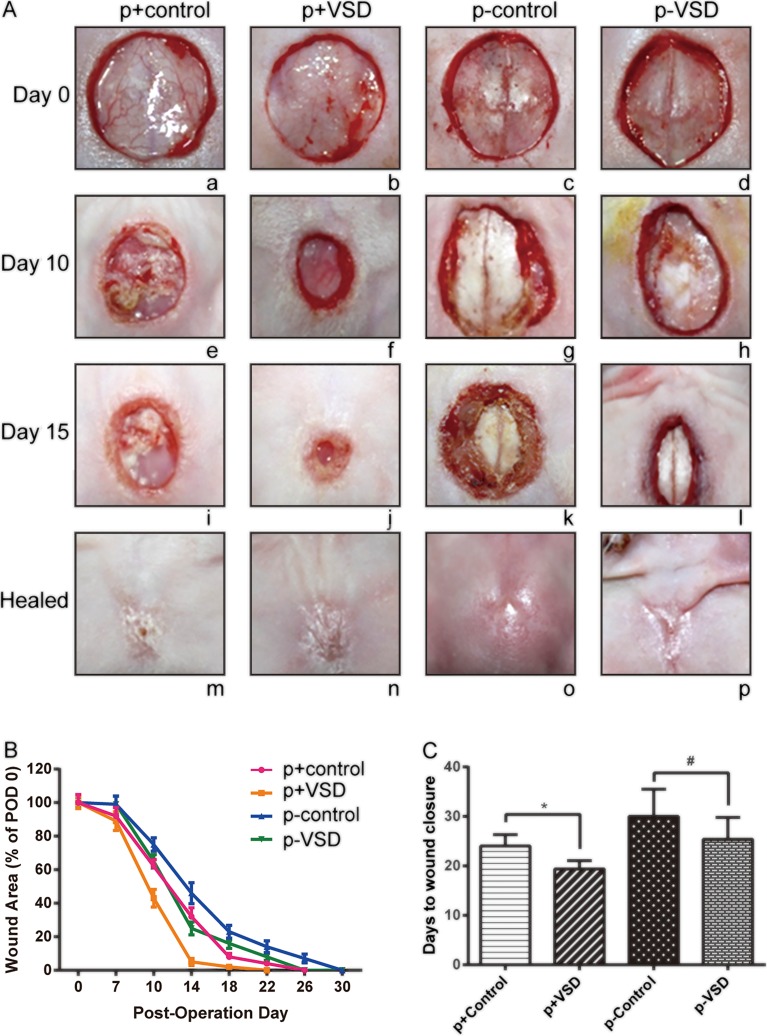
VSD therapy accelerated wound healing. *A*, Effect of VSD on the gross appearance of wound healing on post-operative days (POD) 0, 10, 15 and after healing, in skull exposure wounds in rabbits. *B*, Wound closure rate was assessed based on wound size measurements. p+: periosteum-intact; p–: periosteum-lacking; Control: traditional dressing therapy; VSD: vacuum sealing drainage. Data are reported as means±SD. *P*<*0.05 compared to p+Control, ^#^P*<*0.05 compared to p–Control (*t*-test).

Whether the wounds were periosteum-intact or periosteum-lacking, the wounds treated with VSD exhibited a significantly faster healing rate than those treated with traditional dressing therapy (Figure 1). During the healing process, wounds of p+control and p–control groups displayed purulent discharge, whereas wounds of p+VSD and p–VSD groups were cleaner. Furthermore, the cortical bone surfaces of periosteum-lacking wounds (i.e., p–control and p–VSD groups) looked smooth and dry, while those of the periosteum-intact wounds (i.e., p+control and p+VSD groups) appeared bright red and moist (Figure 1A).

Application VSD significantly accelerated wound healing at each time point of POD 7 to POD 15 compared with utilization of traditional dressing. The periosteum-intact group was also associated with more desirable healing rate compared to the periosteum-lacking group, though eventually the wound healing rates were all shown as 100% (Figure 1B).

It took only 19.40±1.65 days for wounds in the p+VSD group to heal completely, which was much shorter than 24.00±2.31 days in the p+control group (P<0.05). Obviously, the periosteum-lacking wounds demanded a longer healing period, with 30.00±5.50 days in the p–control group. Nonetheless, p–VSD shortened the wound healing time to 25.40±4.43 days (Figure 1C). Interestingly, there was no significant difference of healing time between p–VSD and p+control groups (P*>*0.05).

### Histological observations

**Figure 2.**
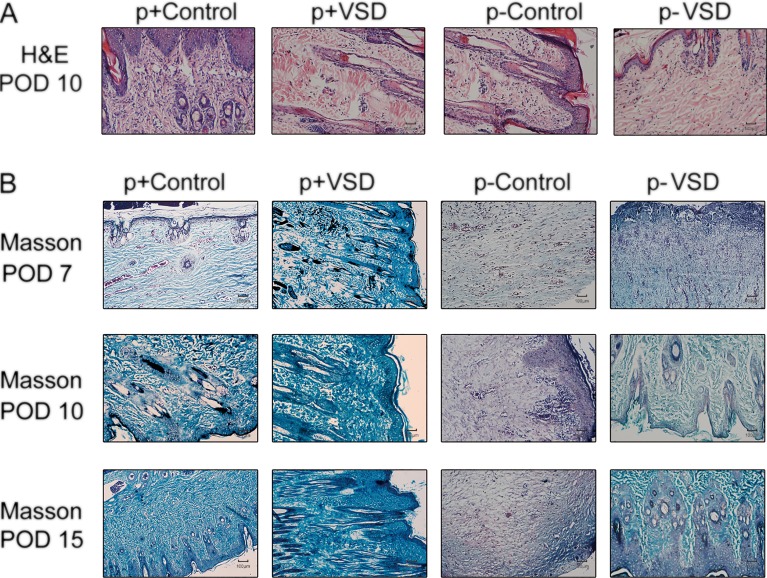
Histological observations of the wounds at different post-operative days (POD). *A*, Slides subjected to H&E staining. The vacuum sealing drainage (VSD)-treated groups had low levels of inflammatory cells infiltration. *B*, Collagen fibers evaluated using Masson's trichrome stain (magnification ×200). VSD-treated groups demonstrated greater collagen organization compared to the corresponding Control group at all time points. p+: periosteum-intact; p–: periosteum-lacking.

In accordance with results of HE staining, wounds treated with traditional dressing therapy showed stronger inflammatory responses and more poorly re-epithelialized epidermis than wounds treated with VSD on POD 10 (Figure 2A). Improved collagen organization was observed in the VSD-treated group as early as POD 7, compared with traditional dressing therapy groups. In spite of the time points, the VSD-treated groups presented more well-organized collagen fibers and faster fibroblast proliferation than the traditional dressing therapy-treated groups (Figure 2B).

### Determination of hydroxyproline content

**Figure 3.**
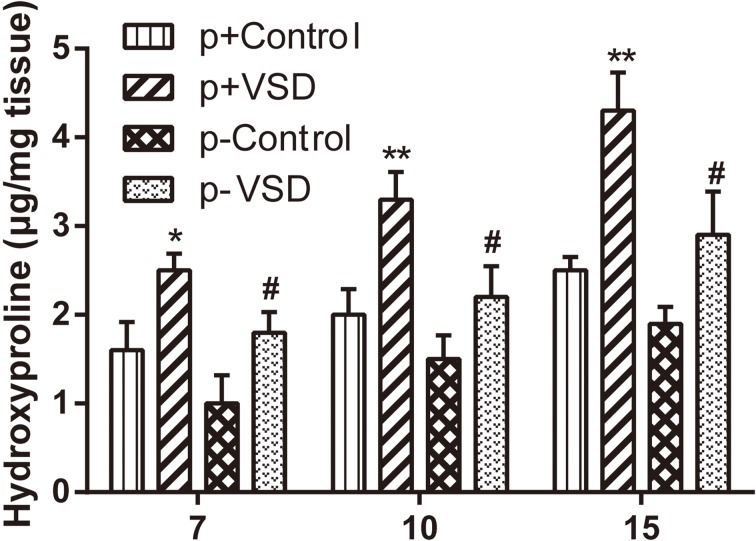
Quantification of hydroxyproline content during the course of wound healing, on post-operative days 7, 10, and 15. The vacuum sealing drainage (VSD)-treated groups had significantly higher levels of hydroxyproline content than controls (traditional treatment). p+: periosteum-intact; p–: periosteum-lacking. Data are reported as means±SD. *P<0.05, **P<0.01 compared to p+Control, ^#^P*<*0.05 compared to p–control.

VSD treatments generally induced higher levels of hydroxyproline than traditional treatment on POD 7, 10 and 15 (P*<*0.05). The hydroxyproline level of the p+VSD group peaked, while the p–control group bottomed (Figure 3). During the period from POD 7 to POD 15, hydroxyproline content showed a significant increasing trend after treatment with VSD (P<0.05), yet no significant differences of hydroxyproline levels were found after traditional treatments (P>0.05).

### VSD-treated wounds showed increased blood vessels

**Figure 4.**
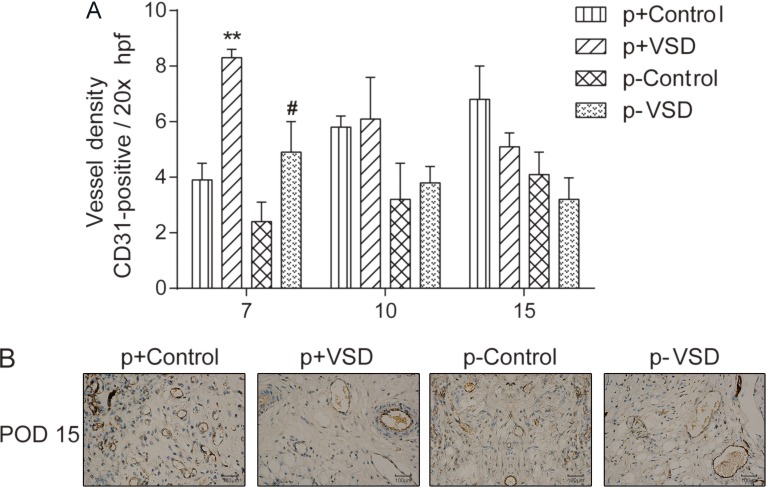
*A*, Microvessel density in tissues during the course of wound healing (post-operative days (POD) 7, 10, and 15). *B*, Vacuum sealing drainage (VSD)-treated wounds had larger-caliber, and more well-developed vessels compared to the traditional dressing wounds (Control), by immunostaining of CD31 on POD 15 (magnification 200×). Data are reported as means±SD. **P*<*0.01 compared to p+Control, ^#^P*<*0.05 compared to p–control. p+: periosteum-intact; p–: periosteum-lacking.

The wounds treated with VSD were correlated with significantly higher vessel density than those treated with traditional dressing on POD 7 (P<0.05; Figure 4A). Similarly, the VSD-treated wounds possessed larger calibers and more well-developed vessels, compared with traditionally treated wounds on POD 10 and POD 15 (Figure 4A and B). From POD 7 to POD 10, a significant increasing tendency of vessel density was only found in wounds of the p+control group (P<0.05).

## Discussion

It is widely known that wound surfaces, though receiving treatments of debridement and active anti-infection, are still readily subjected to severe infection. For example, parental antibiotics usually do not reach effective bactericidal concentration within the local wound microenvironment.

The VSD technique has been expanded dramatically since it was popularized in 1997 by Argenta and Morykwas (2) and further studied in 2010 by Kairinos et al. (13). The application of VSD has been widely recognized as effective in treating soft tissue trauma, however, usage of VSD for treatment of bone-exposed wounds has rarely been comprehensively and thoroughly investigated. For instance, skin graft combined with VSD has been employed to treat large soft tissue defects and bone exposure in the lower leg, which might eliminate the need for amputation and complex surgeries (1). However, that research was limited to the clinical setting, and we designed rabbit models to explore the underlying mechanisms of VSD therapy. Although the rabbit bone-exposed wound is a study model easy to reproduce and inexpensive, the size of the wound is finite. Thus, we would require larger animal models to investigate whether VSD therapy could promote wound healing without skin grafting and skin flap transplantation (14).

It was documented that VSD therapy could improve removal of edema fluid, blood flow, granulation tissue formation and bacterial clearance from wounds (10). Our study consistently showed that wounds undergoing treatment with VSD were clean and without exudate. Moreover, the periosteum (osteogenic tissues surrounding bones) contains multiple stem cells that secrete large quantities of VEGF and BMP2, thereby playing critical roles in boosting bone healing (15–17). In our investigation, the wound healing time showed no notable distinction between p–VSD and p+control groups, further suggesting that VSD therapy could enhance wound healing. The fact that VSD-treated wounds had little inflammatory cell infiltration and well-organized collagen fibers also confirms that VSD therapy could inhibit inflammatory responses and could enhance formation of epidermis.

Hydroxyproline is as a basic constituent of collagen structure, implying that its increased concentration might contribute to improved wound healing activity (18,19). Our results also showed that VSD therapy augmented hydroxyproline concentration both in the periosteum-lacking and periosteum-intact groups, and p+VSD group possessed the highest level of hydroxyproline. In fact, hydroxyproline was crucial at specific moments of healing, for it maintained the equilibrium between synthesis and degradation of extracellular matrix proteins, achieving remodeling of the wounds and reduced formation of scars (20).

Rat wounds treated with negative pressure showed relatively high MVD on day 3, which could be due to the negative pressure functioning as promotor of angiogenesis (21). Also with a rodent model, Jacobs et al. (10) concluded that treatment with vacuum would advance formation of blood vessels by POD 3. In our study, VSD treatment increased blood vessel density in the early days of wound healing through increasing cell number or improving cell quality.

In conclusion, VSD promoted the healing of bone-exposed and periosteum-lacking wounds in a rabbit model by increasing hydroxyproline content and MVD, reducing inflammation, and inducing ordered collagen arrangements.
